# Assessment of Oral Lesions With Tobacco Usage: A Cross-Sectional Clinicopathological Study in Sri Ganganagar, Rajasthan, India

**DOI:** 10.7759/cureus.33428

**Published:** 2023-01-05

**Authors:** Karthikeyan Ramalingam, Murugesan Krishnan, Senthilmurugan Mullainathan, Arjun Sahuwala, Gurveen Chawla, Gheena S

**Affiliations:** 1 Oral Pathology and Microbiology, Saveetha Dental College and Hospital, Chennai, IND; 2 Oral and Maxillofacial Surgery, Saveetha Dental College and Hospital, Chennai, IND; 3 Oral Pathology, Surendera Dental College and Research Institute, Sri Ganganagar, IND; 4 Oral and Maxillofacial Pathology, Saveetha Dental College and Hospital, Chennai, IND

**Keywords:** malignant transformation, toluidine blue, oral lesions, biopsy, oral cancer, oral potentially malignant disorders, india, rajasthan, tobacco chewing, tobacco smoking

## Abstract

Background

Tobacco usage in the form of smoking or chewing has increased the risk of oral potentially malignant disorders (OPMDs) and oral cancer. These deleterious habits are also related to changes in dentition and the oral mucosa.

Aim

The aim of our study was to evaluate the oral changes associated with tobacco usage among residents of Sri Ganganagar.

Materials and methods

This study was conducted among the residents of Sri Ganganagar, Rajasthan, India, using stratified cluster random sampling, prestructured questionnaires, and detailed oral examination. A total of 100 patients with a previous history of tobacco usage were enrolled in this study after obtaining informed consent. Age- and gender-matched controls were also evaluated to correlate the findings. Clinical details were documented, including the Oral Hygiene Index-Simplified (OHI-S), Decayed-Missing-Filled Teeth (DMFT) index, Community Periodontal Index (CPI), loss of attachment, dental findings, and oral mucosal changes. Suspicious lesions were stained with toluidine blue, and a biopsy was performed for histopathological evaluation. The tabulated results were statistically analyzed using the Statistical Package for the Social Sciences (SPSS) version 21.0 (IBM SPSS Statistics, Armonk, NY, USA) for significance.

Results

Attrition, abrasion, and erosion of teeth were more frequent in tobacco users than in controls. Smoker’s palate, tobacco pouch keratosis, and leukoplakia were commonly noted mucosal lesions. The mean values of the parameters of the DMFT score (3.560), CPI score (2.190), and loss of attachment score (0.542) were higher among tobacco users, and it was statistically significant (P value < 0.05). Out of 100 patients, 17 had suspicious lesions. It included seven cases of oral submucous fibrosis (OSMF), two cases of tobacco pouch keratosis, and eight cases of leukoplakia. Toluidine blue staining and biopsy were performed. Histopathological examination of suspicious lesions revealed hyperkeratosis, various grades of epithelial dysplasia, and differing inflammatory responses. Out of 17 biopsied cases, there were two cases of hyperkeratosis with severe epithelial dysplasia, four cases of hyperkeratosis with moderate epithelial dysplasia, two cases of hyperkeratosis with mild dysplasia, two cases of superficially invasive squamous cell carcinoma, five cases of advanced OSMF, and two cases of moderately advanced OSMF.

Conclusion

Tobacco usage produces visible changes in dentition and latent alterations in the oral mucosa. Suspicious lesions should always be referred for histopathological examination to identify oral potentially malignant disorders and oral cancer so that prompt treatment could be initiated. Patient education is mandatory to avoid the usage of tobacco in any form.

## Introduction

In a high-risk country like India, oral cancer is in first place among males and third place among females. Its incidence is very high (20 cases in 100,000) [1]. The World Health Organization (WHO) has reported that approximately 13% of all deaths in India could be attributed to tobacco usage and that it accounts for more than 1.5 million deaths annually. Tobacco consumption varies between urban and rural populations with differing intensity among socioeconomic groups [2].

Tobacco, areca nut, and alcohol usage are the main etiological factors for oral cancer [3]. Oral potentially malignant disorders (OPMDs) comprising diverse oral mucosal lesions have an increased risk of malignant transformation than normal. The frequently encountered OPMD includes leukoplakia, proliferative verrucous leukoplakia, erythroplakia, oral submucous fibrosis (OSMF), lichen planus, and oral lichenoid lesions [4].

The prevalence of OPMD has been studied across the Indian subcontinent [4-15]. Karthikeyan et al. have also studied the changes in normal oral mucosa due to smoking and chewing tobacco [11]. A literature search revealed limited studies on Rajasthani populations. Hence, our study was done to identify the oral changes associated with tobacco usage among residents of Sri Ganganagar, Rajasthan, India.

## Materials and methods

This was a cross-sectional clinicopathological study conducted among the residents of Sri Ganganagar, Rajasthan, India, using prestructured questionnaires and detailed oral examination. Stratified cluster random sampling was taken from four zones: North, South, East, and West zones of Sri Ganganagar. The study was conducted in accordance with the Declaration of Helsinki, and ethical clearance was obtained (IHEC/SDC/PhD/OPATH-2212/22/001).

Inclusion criteria were both males and females aged above 15 years who are ready to disclose their habits and consent for oral examination and biopsy if needed. They should have had the tobacco habit for a minimum period of 12 months and still actively continuing the habit. Exclusion criteria were subjects below 15 years of age, subjects who did not want to disclose their deleterious habits, and who did not consent to oral examination and biopsy.

A total of 100 patients with a previous 12-month history of tobacco usage were enrolled in the study after obtaining informed consent. Age- and gender-matched controls were also evaluated to correlate the clinical findings. Complete details of the patients were recorded, including age, sex, tobacco habit (smoking/chewing/both), alcohol consumption, and previous treatment if any.

The oral examination was performed with mouth mirrors under proper illumination. Dental findings, the Oral Hygiene Index-Simplified (OHI-S), the Decayed-Missing-Filled Teeth (DMFT) index, the Community Periodontal Index (CPI), loss of attachment, and presence of oral mucosal changes, if any, were documented. The sites of the lesions were recorded as buccal mucosa, tongue, floor of the mouth, alveolar ridge, gingivobuccal sulcus, retromolar trigone, palate, lip, and pharynx/peritonsillar region.

Suspicious lesions were photographed and stained with toluidine blue, a biopsy was performed, and histopathological evaluation was done using routine hematoxylin and eosin (H&E) staining. The epithelium and connective tissue were assessed for the nature of keratinization, features of epithelial dysplasia (both cellular changes and architectural changes), nature of underlying stroma, and inflammatory components. The basement membrane integrity was evaluated for any discontinuity or breach.

The tabulated results were statistically analyzed using the Statistical Package for the Social Sciences (SPSS) version 21.0 (IBM SPSS Statistics, Armonk, NY, USA) for significance. A P value of <0.05 was considered to be statistically significant.

## Results

A total of 100 patients residing in Sri Ganganagar, Rajasthan, India, were included in the study. The age range of the included samples was 15-80 years, with a mean age of 47.5 years. Of the patients, 63% were males and 37% were females.

Tobacco was used both in smoked and smokeless forms. Bidi and cigarette smoking were identified in 51% of the study group. Tobacco chewing with commercially available sachets was noted in 49% of the study group. In our study, patients with tobacco smoking and tobacco chewing showed oral lesions affecting the teeth and mucosa.

**Figure 1:**
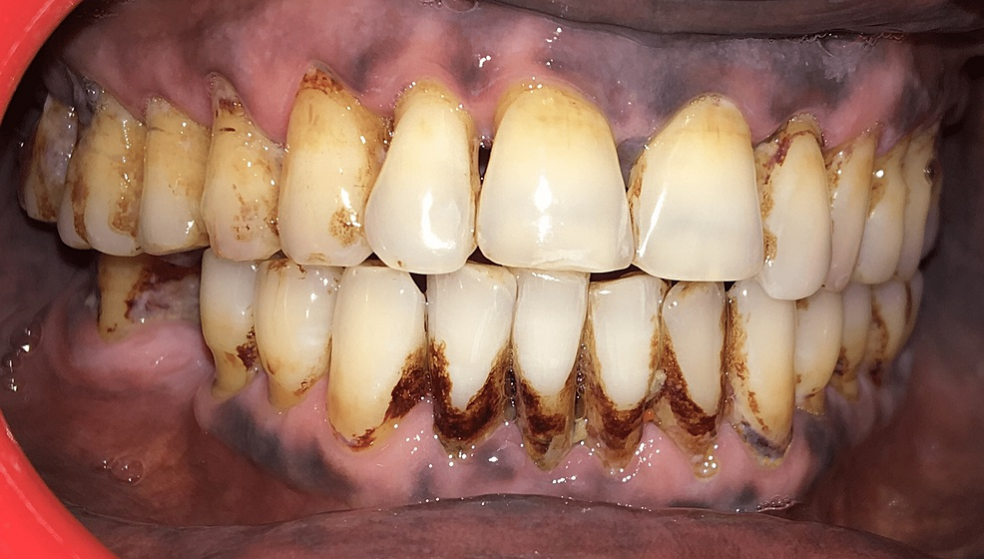
Clinical picture Extrinsic dental stains and poor oral hygiene in a tobacco user

Overall oral hygiene was poorer in tobacco users than in the control group. The mean OHI-S score was 4.4 among tobacco users, while it was 1.4 in the control group (Figure 1).

**Figure 2:**
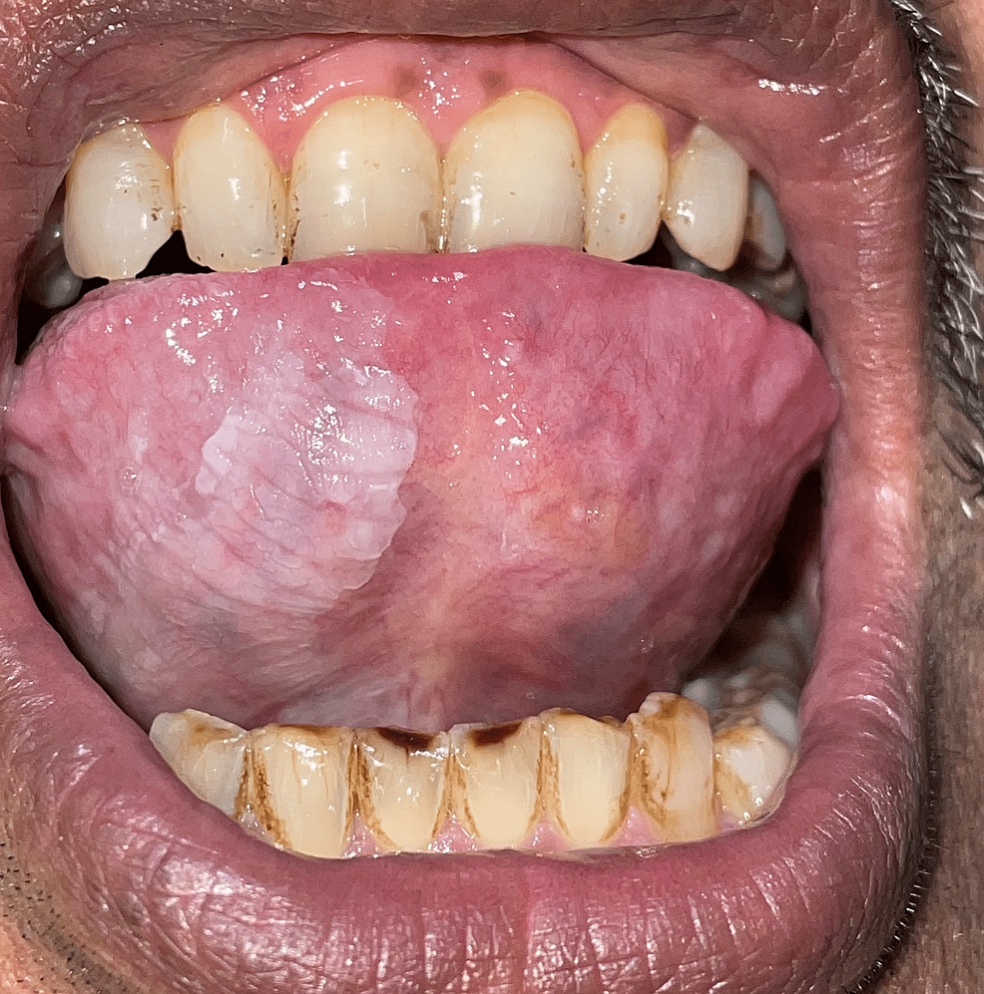
Clinical picture Leukoplakia on the right ventral surface of the tongue along with attrition, staining, and erosion of mandibular anterior teeth

Attrition (46%), abrasion (34%), and erosion (28%) of teeth were more frequent in tobacco users, especially tobacco chewers, than in control groups. Extrinsic stains (92%) were more frequent among tobacco users (Figure 1 and Figure 2).

The mean DMFT score was higher among tobacco users (3.560), whereas it was only 1.45 in the control group. The mean CPI score was higher among tobacco users (2.190) than the control group (1.241). The mean loss of attachment score was higher among tobacco users (0.542) than the control group (1.241). These values were statistically significant with a P value of <0.05.

**Figure 3:**
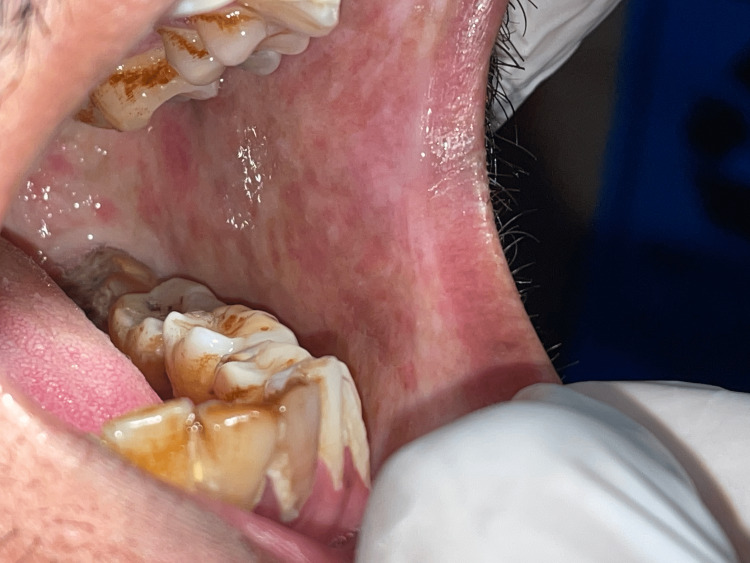
Clinical picture Fibrotic bands of the left buccal mucosa, dental plaque, and debris with staining noted in a patient with oral submucous fibrosis

Twenty-eight patients showed oral mucosal lesions. The sites involved were the palate (n = 11), buccal mucosa (n = 15), and tongue (n = 2). Oral mucosal lesions including smoker’s palate (11%), tobacco pouch keratosis (2%), leukoplakia (8%) (Figure 2), and oral submucous fibrosis (OSMF) (7%) (Figure 3) were commonly noted among tobacco users (Table 1).

**Table 1:** Types of oral mucosal lesions Case distribution of different oral mucosal lesions OSMF: oral submucous fibrosis

Type of oral mucosal lesions	Number of cases (n = 28)
Leukoplakia	8
OSMF	7
Tobacco pouch keratosis	2
Smoker’s palate	11

Out of 100 patients, 17 had suspicious lesions with toluidine blue staining, and a biopsy was performed. It included seven cases of OSMF, two cases of tobacco pouch keratosis, and eight cases of leukoplakia.

Histopathological examination of the suspicious lesions revealed hyperkeratosis, various grades of epithelial dysplasia, carcinomatous change, and differing inflammatory responses (Table 2).

**Table 2:** Histopathological findings Various histopathological findings of the 17 biopsies OSMF: oral submucous fibrosis

Histopathology	Number of patients (n = 17)
Hyperkeratosis with severe epithelial dysplasia	2
Hyperkeratosis with moderate epithelial dysplasia	4
Hyperkeratosis with mild epithelial dysplasia	2
OSMF with well-differentiated squamous cell carcinoma	1
OSMF with moderate epithelial dysplasia	2
OSMF with mild epithelial dysplasia	1
Superficially invasive squamous cell carcinoma	2
OSMF	2

**Figure 4:**
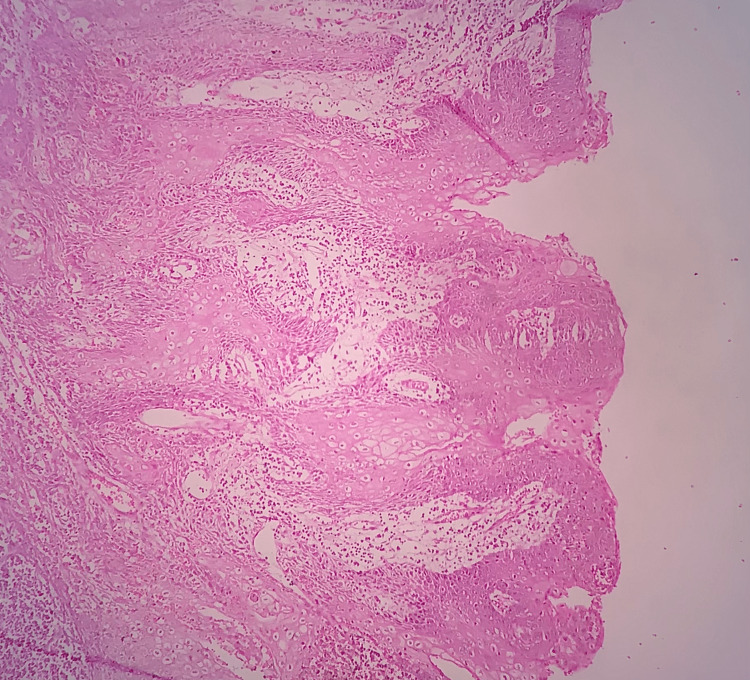
Photomicrograph Dysplastic epithelium along with early invasive squamous cell carcinoma (H&E: 40×) H&E: hematoxylin and eosin

Out of the 17 biopsied cases, two cases showed hyperkeratosis with severe epithelial dysplasia, four cases showed hyperkeratosis with moderate epithelial dysplasia, two cases showed hyperkeratosis with mild dysplasia, two cases showed superficially invasive squamous cell carcinoma (Figure 4), five cases showed advanced OSMF, and two cases showed moderately advanced OSMF.

**Figure 5:**
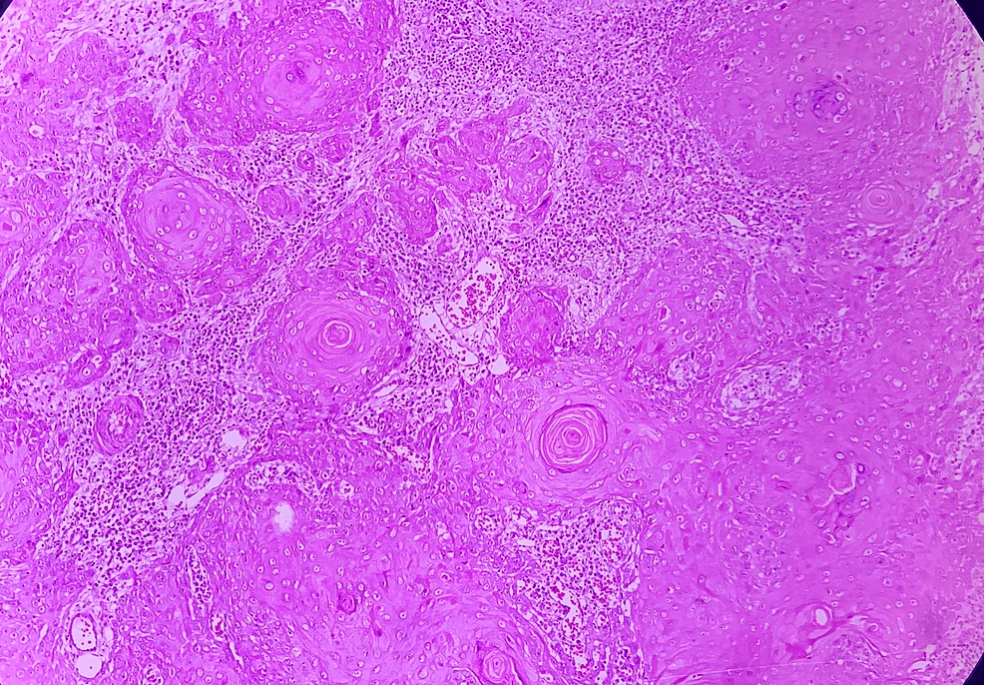
Photomicrograph Malignant epithelial islands with keratin pearl formation in well-differentiated squamous cell carcinoma (H&E: 10×) H&E: hematoxylin and eosin

**Figure 6:**
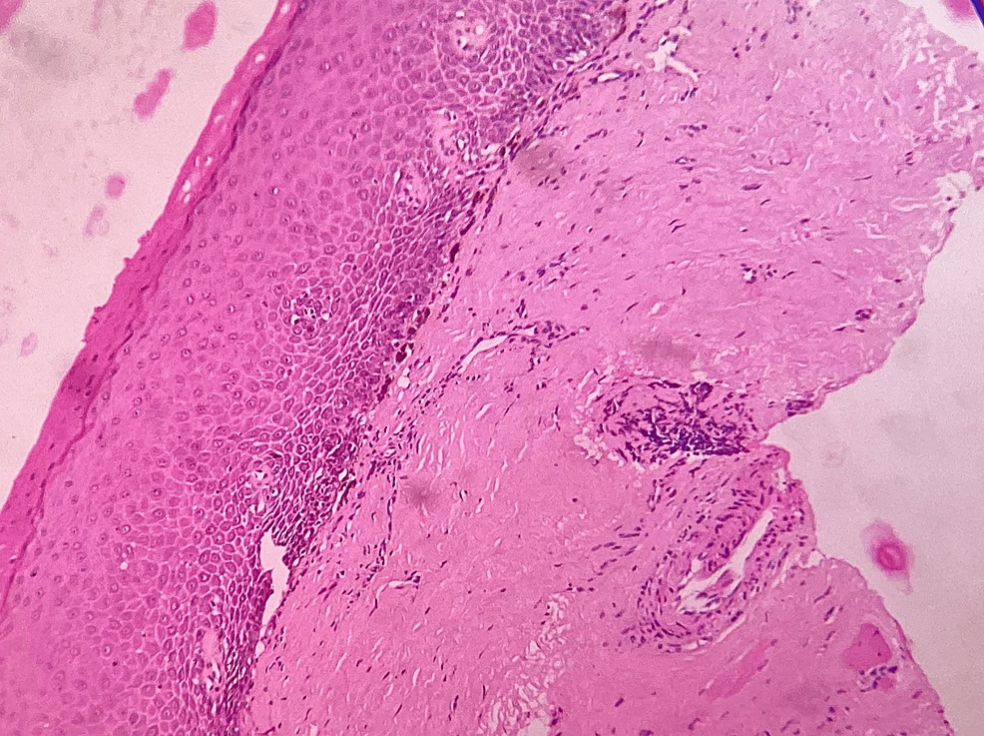
Photomicrograph Dysplastic epithelium with hyalinized connective tissue in oral submucous fibrosis (H&E: 40×) H&E: hematoxylin and eosin

We also found that out of five advanced OSMF cases, one case also showed well-differentiated squamous cell carcinoma (Figure 5), one case had moderate epithelial dysplasia, and one case had mild epithelial dysplasia. Out of two moderately advanced OSMF cases, one case also had moderate epithelial dysplasia (Figure 6).

## Discussion

Oral health is influenced by deleterious oral habits such as tobacco smoking and chewing. The oral mucosa responds by increasing epithelial thickness and keratinization. Tobacco in any form contains dangerous nitrosamines that are implicated in carcinogenesis [6]. Areca nut is also highly addictive, and it contains carcinogenic alkaloids [16]. Based on the type, duration, and frequency of tobacco usage, dysplastic changes begin within epithelial cells and could progress to OPMD and ultimately into oral cancer [6].

Kumbhalwar et al. have reported in their meta-analysis that the majority of the prevalence studies have been performed in southern states of India [2]. Most of the studies were performed in specific groups such as fishermen or urban or rural populations or patients reporting to a dental college. Our study was conducted in Sri Ganganagar, Rajasthan, which comes under the semi-urban subtype, and only residents were included.

Krishna Priya et al. reported that 42.4% of oral mucosal lesions are associated with tobacco and alcohol habits [9]. Kumbhalwar et al. have reported a 5%-6% of leukoplakia and 4%-6% of OSMF in their systematic review and meta-analysis [2]. In our study, 8% were leukoplakia cases and 7% were OSMF cases.

A biopsy is considered the gold standard for diagnosis, but the clinician should be able to differentiate OPMD from reactive or inflammatory diseases. In vivo staining with toluidine blue, Lugol’s iodine, crystal violet, acetic acid staining, oral exfoliative cytology, autofluorescence, and chemiluminescence are utilized for such situations [6,12,17,18]. We have performed a biopsy for all suspicious lesions identified in our study.

Vijayakumar et al. reported 92.6% sensitivity and 67.9% specificity of toluidine blue staining [17]. The limitations of toluidine blue staining are that hyperkeratotic lesions may show false-negative results and ulcerated lesions can show false-positive results. Hence, the combination of toluidine blue staining with histopathological confirmation will improve its diagnostic validity [17,18]. In our study, all cases were subjected to toluidine blue staining and were histopathologically evaluated.

Samatha et al. reported that 50 out of 76 tobacco-related lesions showed dysplastic features [12]. In our study, 12 of 17 cases had epithelial dysplasia. Muthukrishnan et al. reported that OPMD has a higher risk of the occurrence of oral cancer in the lip and oral cavity [19]. Iocca et al. reported that 9.5% of leukoplakia cases and 5.2% of OSMF cases showed malignant transformation [4]. In our study, three cases showed malignant transformation, of which two were leukoplakia and one was OSMF.

Posttreatment complications are observed after surgical intervention, radiotherapy, and chemotherapy [20]. Newer techniques with nanoparticles and natural active ingredients could play a potential role in the future [21-24]. Increasing trends of oral cancer incidence among Indian men and women could be mitigated by widespread public awareness campaigns against the ill effects of tobacco usage, thereby reducing the risk factors associated with oral carcinogenesis [25,26].

Sarode et al. reported that OSMF showing basal cell layer hyperplasia, abnormal superficial mitosis, alterations in nuclear/cytoplasmic ratio, and hyperchromasia have increased risk for malignant transformation [27]. Such patients should be kept under strict follow-up for early identification and prompt management.

Limitations

A limited sample size was included in our study, as Sri Ganganagar is not densely populated like other major cities of Rajasthan. A detailed clinical history and further investigations were not possible due to ethnic variations and cultural inhibitions. Stratification of patients based on deleterious habits and site-wise distribution of oral lesions will help in the implementation of appropriate strategies.

## Conclusions

Our study is the pilot initiative to report oral lesions associated with tobacco usage in Sri Ganganagar, the northwestern region of the Indian subcontinent. It is surprising to see that this sparsely populated region of Thar Desert has a dedicated free train service for cancer patients to get treatment in Bathinda, Punjab, and Bikaner, Rajasthan. Our findings show that tobacco usage is widely seen in both smoked and chewable forms. Oral mucosal lesions including OPMD and dental lesions such as extrinsic stains, attrition, abrasion, and erosion are evident in this population. It is shocking to see that many OPMDs showed features of severe dysplasia, early invasive squamous cell carcinoma, and well-differentiated squamous cell carcinoma.

Hence, regular dental checkups should be emphasized to identify potential risk factors, educate the public, and minimize the morbidity associated with oral cancer. Multicenter studies within Rajasthan along with longitudinal follow-up will provide valuable information about tobacco usage, associated oral lesions, its rate of progression, and malignant conversion ratios. Due to regional variations in diet and climatic conditions, this study will also help in planning public awareness campaigns against tobacco usage.
